# The Molecular Role of Polyamines in Age-Related Diseases: An Update

**DOI:** 10.3390/ijms242216469

**Published:** 2023-11-17

**Authors:** Guadalupe Elizabeth Jimenez Gutierrez, Fabiola V. Borbolla Jiménez, Luis G. Muñoz, Yessica Sarai Tapia Guerrero, Nadia Mireya Murillo Melo, José Melesio Cristóbal-Luna, Norberto Leyva Garcia, Joaquín Cordero-Martínez, Jonathan J. Magaña

**Affiliations:** 1 Laboratorio de Medicina Genómica, Instituto Nacional de Rehabilitación Luis Guillermo Ibarra Ibarra, Mexico City 14389, Mexico; gejimenez@inr.gob.mx (G.E.J.G.); fabiola_borbolla@comunidad.unam.mx (F.V.B.J.); german.munoz@cinvestav.mx (L.G.M.); ytapia@inr.gob.mx (Y.S.T.G.); nmurillo@inr.gob.mx (N.M.M.M.); nleyva@inr.gob.mx (N.L.G.); 2 Departamento de Farmacia, Facultad de Química, Universidad Nacional Autónoma de México, Ciudad Universitaria, Mexico City 04510, Mexico; 3 Departamento de Farmacia, Escuela Nacional de Ciencias Biológicas del Instituto Politécnico Nacional, Mexico City 07738, Mexico; jcristoball@ipn.mx; 4 Laboratorio de Bioquímica Farmacológica, Departamento de Bioquímica, Escuela Nacional de Ciencias Biológicas, Instituto Politécnico Nacional, Mexico City 11340, Mexico; 5 Department of Bioengineering, Escuela de Ingeniería y Ciencias, Tecnologico de Monterrey, Campus Ciudad de México, Mexico City 14380, Mexico

**Keywords:** polyamines, spermidine, spermine, putrescine, aging, age-related diseases, aging biomarkers

## Abstract

Polyamines (Pas) are short molecules that exhibit two or three amine groups that are positively charged at a physiological pH. These small molecules are present in high concentrations in a wide variety of organisms and tissues, suggesting that they play an important role in cellular physiology. Polyamines include spermine, spermidine, and putrescine, which play important roles in age-related diseases that have not been completely elucidated. Aging is a natural process, defined as the time-related deterioration of the physiological functions; it is considered a risk factor for degenerative diseases such as cardiovascular, neurodegenerative, and musculoskeletal diseases; arthritis; and even cancer. In this review, we provide a new perspective on the participation of Pas in the cellular and molecular processes related to age-related diseases, focusing our attention on important degenerative diseases such as Alzheimerߣs disease, Parkinsonߣs disease, osteoarthritis, sarcopenia, and osteoporosis. This new perspective leads us to propose that Pas function as novel biomarkers for age-related diseases, with the main purpose of achieving new molecular alternatives for healthier aging.

## 1. Introduction

People around the world are living longer. In 2021, the World Health Organization (WHO) estimated that by 2030, one in six people will be 60 years of age or older. The number of people aged 80 years or older will triple from 2020 to 2050, reaching 426 million [1]. The epidemiological evidence has demonstrated the effects of age-related challenges, especially in terms of the burdens on economic growth and healthcare systems. These systems are currently facing the challenges of complex health conditions and age-related diseases [2,3]. Aging is characterized by the progressive decline in physical and psychological capacities that occurs concomitantly with the onset of chronic degenerative diseases. In this context, the search for molecular targets that could serve as more effective adjuvants in anti-aging therapies or in the treatment of age-related diseases is becoming the number-one priority. The main age-related diseases are cardiovascular, neurodegenerative, and musculoskeletal diseases; arthritis; and cancer [4]; furthermore, according to the Global Burden of Disease, Injuries, and Risk Factors Study in 2017, 31.4% of all diseases were found to be associated with age [5]. Interestingly, in recent years, a plethora of cellular and molecular hallmarks of aging have been described, such as genomic instability, a loss of proteostasis, deregulated nutrient sensing, mitochondrial dysfunction, cellular senescence, and altered intracellular communication, among others [6,7]. Organ and tissue analyses of these molecular changes might provide accessible markers of aging stages and progression and help to identify novel geroprotective compounds. Possible clinical interventions and treatments include strategies such as lifestyle interventions (diet, exercise, and weight loss), pharmacological approaches (antioxidants and senolytic, senomorphic, and anti-inflammatory drugs), modulating the gut microbiota, cell transplantation, gene therapy, and immunotherapy [8]. All of these strategies have the final goal of achieving healthy aging and longevity.

Polyamines (Pas) are interesting candidates in the search for anti-aging adjuvants because of their interactive capacity to participate in essential life functions such as proliferation, differentiation, apoptosis, lipid metabolism, and aging [9]. Pas are well-known molecules that were discovered more than 300 years ago; they are present in all eukaryotes and in the majority of prokaryotes [10]. The main mammalian Pas, putrescine (PUT), spermine (SPM), and spermidine (SPD), are short, positively charged molecules at a physiological pH. They are composed of two or more amine groups that are ubiquitously expressed and interact with some of the major classes of negatively charged regions of biomolecules such as nucleic acids and proteins [11]. Our objective is to provide a new perspective on the participation of Pas in the cellular and molecular processes linked to age-related diseases, leading us to propose new molecular alternatives for healthier aging.

## 2. Biosynthesis of Pas

There are three principal sources of Pas in organisms: dietary intake, cellular synthesis, and gut microbiota synthesis [12,13,14]. Early reports hypothesized that extracellular Pas enter cells through different systems: the heparin sulfate and glypican 1 (GPC1) system, which transports spermine; endocytosis, mediated by Caveolin-1; and the exportation of putrescine by the transporter SLC3A2 [15]. Recent studies have shown that in different cells and tissues, the transport of spermine and spermidine is executed through polyspecific organic cation transporters 1, 2, and 3 (OTC-1-3) [16,17].

**Figure 1:**
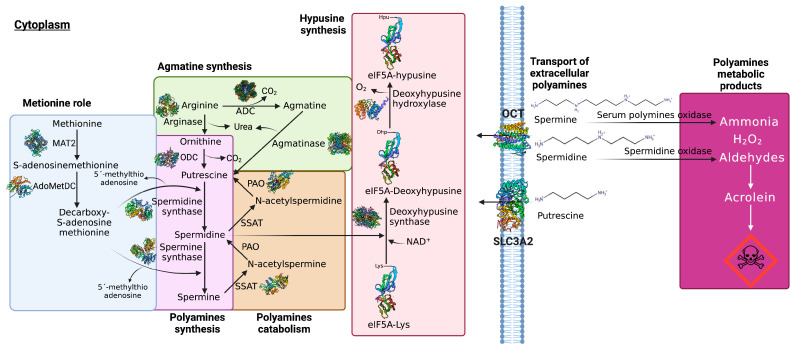
PA transport and metabolism. GPC1 transports spermine, putrescine is exported by the transporter SLC3A2, and spermine and spermidine are transported through OCT–1–3. In the first stage of PA synthesis, ornithine and agmatine are synthetized from arginine catalyzed by arginase and ADC, respectively. Agmatine can be transformed into putrescine and urea by agmatinase. Ornithine is then decarboxylated by ODC to synthetize putrescine and urea. Subsequently, putrescine is converted into spermidine and spermidine is converted into spermine by the action of spermidine synthetase and spermine synthetase, respectively. Spermidine is cleaved and transferred by DHS to eIF5A-Lys to catalyze the interconversion of deoxyhypusine, which is subsequently hydroxylated by DOHH to produce hypusine. In a second stage, dcAdoMet suffers a decarboxylation catalyzed by S–adenosine methionine decarboxylase (pyruvoyl), producing S–adenosine methionine–3–aminopropyl methyl sulfonate. The catabolism pathway of PA successively converts spermine into spermidine and spermidine into putrescine through the acetylated forms of PA (N–acetylspermine and N–acetylspermidine) catalyzed by SSAT and PAO. The degradation of PA leads to the production of highly toxic intermediaries such as aldehydes, peroxides, and ammonia. In particular, aldehydes are extremely reactive and degrade spontaneously, producing acrolein, a highly toxic compound.

The cellular synthesis of Pas arises from the amino acids arginine, ornithine, and methionine, and the first stage of synthesis includes the production of ornithine and agmatine from arginine catalyzed by arginase (EC 3.5.3.1) and arginine decarboxylase (ADC) (EC 4.1.1.19), respectively. Alternatively, the agmatine could be transformed into putrescine and urea by agmatinase (EC 3.5.3.11). Once ornithine is obtained, it is then decarboxylated by ornithine decarboxylase (ODC) (EC 4.1.1.17) to synthetize putrescine and urea [18]. ODC is considered the rate-limiting factor of PA synthesis; therefore, its inhibition may be a suitable strategy for the treatment of cancer [19,20,21]. Subsequently, putrescine is converted into spermidine and spermidine is converted into spermine via the action of spermidine synthetase (EC 2.5.1.16) and spermine synthetase (EC 2.5.1.22), respectively [22] (Figure 1). Spermidine is also cleaved and transferred by deoxyhypusine synthase (DHS) (EC 2.5.1.46) to eukaryotic translation factor 5A (eIF5A)-Lys to catalyze the interconversion of deoxyhypusine, which is subsequently hydroxylated by deoxyhypusine hydroxylase (DOHH) (EC 1.14.99.29) to produce hypusine. This is considered an unusual amino acid that is essential to the function of eIF5A. The synthesis of hypusine-containing proteins is the most specific post-translational modification known so far, and it is performed enzymatically to complete the maturation of eIF5A [23,24] (Figure 1).

In a second stage, S-adenosylmethionine (dcAdoMet) suffers decarboxylation, catalyzed by S-adenosine methionine decarboxylase (pyruvoyl) (EC 4.1.1.50), producing S-adenosine methionine-3-aminopropyl methyl sulfonate (also known as decarboxylated S-adenosyl-l-methionine), which is an aminopropyl donor used by spermidine synthetase and spermine synthetase to produce spermine and spermidine, respectively. Furthermore, the residual 5ߣ-methylthioadenosin (MTA) can induce apoptosis by itself in abnormal cells [4,22] (Figure 1). The catabolism pathway of PA successively converts spermine into spermidine and spermidine into putrescine through the acetylated forms of PA (N-acetylspermine and N-acetylspermidine) catalyzed by spermine/spermidine N1-acetyltransferase (SSAT) (EC 2.3.1.57) and polyamine oxidase (PAO) (EC 1.5.3.11) [25,26]. Despite these various physiological functions and cellular needs, Pas are toxic to cells, either due to their high concentrations or via their degradation, which leads to the production of highly toxic intermediaries such as aldehydes, peroxides, and ammonia. Notably, aldehydes are extremely reactive and degrade spontaneously, producing acrolein, a highly toxic compound [27] (Figure 1).

## 3. Polyamines as Novel Biomarkers for Age-Related Diseases

Aging is a natural process; it controls numerous biological and genetic events that are the driving force for all age-related diseases. Although most of the existing research has focused on understanding the molecular mechanisms of aging, developing new strategies for the early diagnosis of age-related diseases should be considered a priority to facilitate better interventions. Our review aims to highlight the advantages of using metabolomics as a risk stratification tool for the detection of age-related diseases. The potential benefits of using metabolomics include a simple approach to new biomarkers in biological fluids or tissues and the provision of predictive information for various clinical variables in age-related diseases [28,29].

Because of the plethora of functions of metabolites such as Pas, they can be considered disease indicators. For example, in cancer, they have been widely used in diagnosis and as markers of tumor progression, as cancer patients have elevated levels of polyamines [30,31,32,33,34]. As such, there is more than one reason to consider polyamines as novel biomarkers that could indicate the progression of age-related diseases. For example, the most recent work concerning Pas as potential biomarkers in age-related diseases was performed via a metabolomic analysis using serum from individuals with mild cognitive impairment (MCI) and Alzheimerߣs disease, where differentially disrupted levels of polyamines and their metabolites were evident two years before MCI would be diagnosed as AD, demonstrating their high predictive capacity in the progression to AD, although it is necessary to establish specific conditions that can be reproduced worldwide [35]. Another work group used enzyme-linked immunosorbent assays, showing elevated serum spermidine levels in MCI subjects with underlying AD, again indicating the potential of polyamines as biomarkers for the progression from MCI to AD [36]. For Parkinsonߣs disease, a metabolomic analysis of the plasma of individuals with the disease showed that polyamine-acetylated metabolites, such as N8-acetylspermidine and N-acetylputrescine, were elevated in PD compared to controls, strongly suggesting that Pas function as useful biomarkers for diagnosis and determining the severity of the disease [37].

**Figure 2:**
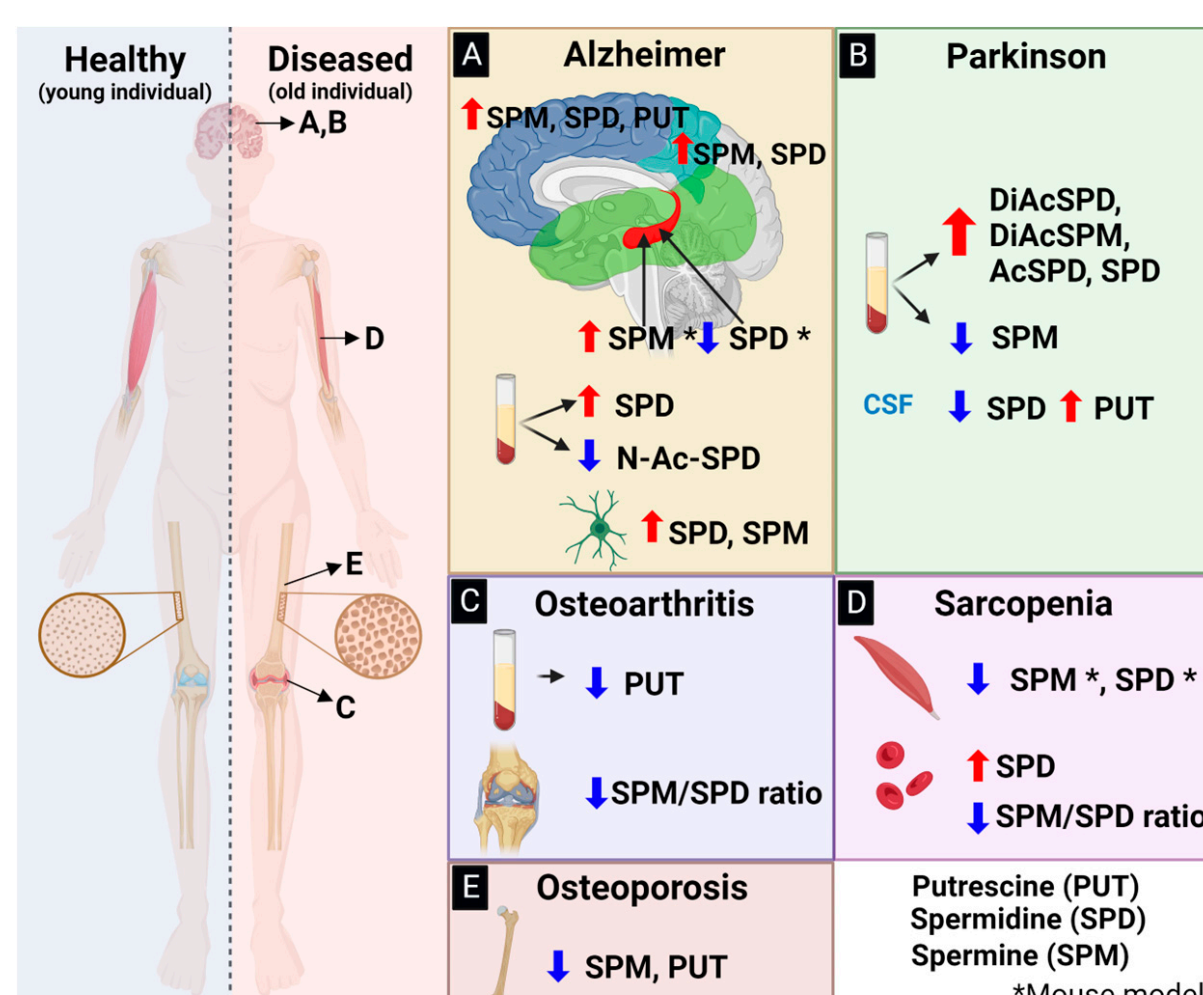
Alterations of polyamine concentration in various age-related diseases. (**A**) Alzheimer’s disease: Predominantly increased polyamines are observed in the brain: SPM, SPD, and PUT increase in the frontal lobe (navy blue); SPM and SPD increase in the parietal lobe (light blue); SPD increases in the temporal lobe (green); and SPM increases while SPD decreases in the hippocampus of mice. In plasma, there is an increase in SPD and a decrease in its acetylated form, N-Ac-SPD. Glial cells show increases in SPD and SPM. (**B**) Parkinson’s disease: Acetylated forms of SPD, SPM, and SPD exhibit higher concentrations, along with an increase in SPD and a decrease in SPM in plasma. There is an increase in SPD and decreases in SPM and PUT in blood. PUT increases and SPD decreases in cerebrospinal fluid. Glial cells exhibit increases in SPD and SPM. (**C**) Osteoarthritis: PUT decreases in plasma, and SPM decreases at the knee. (**D**) Sarcopenia: SPM and SPD decrease in the muscle, while there is an increase in SPD and a decrease in SPM in blood. (**E**) Osteoporosis: There are decreases in SPD and PUT in osteoporotic bones. Labels indicate putrescine (PUT), spermidine (SPD), spermine (SPM), and their acetylated versions (Ac) either at the amino terminus (N-) or di-acetylation (Di). A higher concentration is indicated by a red arrow, while a lower concentration is denoted by a blue arrow, compared to healthy individuals. * Refers to studies using murine models.

There are very few biomarkers corresponding to age-related diseases linked to mobility disabilities, such as osteoarthritis or sarcopenia, and more are needed. A recent study used a metabolomic analysis to detect the systemic changes in the amino acids and polyamines of individuals with severe OA compared to controls after adjusting the bone mass index (BMI); higher levels of spermidine were found, along with a lower ratio of spermine and spermidine, suggesting their potential use in clinical practice (Figure 2 and Table 1) [38,39]. The possibility of using polyamines as biomarkers for sarcopenia is almost a reality; the latest research suggests a progressive decrease in the spermine/spermidine ratio in serum from healthy to sarcopenic subjects [40,41]. The use of metabolomics is emerging in the osteoporosis field [42,43], where the newest evidence highlights the use of the metabolome and its association with fragility fractures; the results have shown higher baseline spermidine levels to be associated with a higher risk of osteoporotic fractures in the Korean community, suggesting a new prognostic biomarker for osteoporosis [44].

In addition to determining the potential role of PAs as novel biomarkers for age-related diseases, it is crucial to establish large-scale biological and statistical validation for clinical practice. This validation should be conducted across a diverse range of cohorts, including multicentric and unselected prospective cohorts of healthy individuals, as well as clinic-based cohorts. The goal is to ascertain whether monitoring fluctuations in PAs, either independently or in combination with other tools and methods, can improve the precision of preventing and diagnosing disease, assessing disease severity, and tracking the progress of age-related diseases.

## 4. Polyamines and Neurodegenerative Diseases

It is known that PAs play essential roles in the central nervous system (CNS). PAs are considered primordial stress inducers because they elicit the polyamine stress response (PSR) in response to various temporary stimuli, such as reactive oxygen species (ROS), heat, ultraviolet light (UV), and even aging. This response offers beneficial effects for survival, but its persistent effect leads to other disorders that contribute to neurodegeneration, commonly with arginine deprivation and increased polyamine levels [61,62], suggesting that PA levels are important for the replication and maintenance of neurons [22]. In the mammalian CNS, PAs play a regulatory role in glutamate receptors and have the potential to modulate N-methyl-D-aspartate (NMDA) receptors. These NMDA receptors are crucial for controlling synaptic plasticity, which, in turn, regulates various neurological functions, including memory [63].

In general, PAs can enhance the opening of channels and regulate glutamate signaling, with an impact on neuronal excitability, memory, and aging [64]. Aging is the principal risk factor for neurogenerative diseases [65], which are believed to share the familiar mechanism of the protein aggregation of diverse misfolded proteins, leading to the degeneration of the CNS. The early detection of neurodegenerative diseases could offer an opportunity for the treatment and prevention of disease progression [66]. In this context, although PAs and their metabolite products have been widely studied in other diseases, there is still the need to understand the connection between the molecular mechanisms of PA metabolism and neurodegenerative diseases in order to identify possible therapeutic approaches for these conditions.

### 4.1. The Role of PAs in Alzheimer’s Disease

It has long been known that the dysregulation of PA metabolism or PAs’ upstream regulators is involved in the neurodegeneration of the CNS [37,45,67]. Metabolic profiles of the brain have shown that PAs have the capacity to bind the amyloid (A) beta peptide (1–40) to promote the classic aggregation of AD, a complex neurodegenerative disease distinguished by progressive memory and cognitive decline, accompanied by alterations in behavior and visuospatial orientation. AD is the most common cause of dementia [68]. As mentioned above, in AD, PA levels are disrupted, even though spermidine and spermine significantly increase their levels; however, the possible mechanism has not been completely elucidated [45,47,49]. Nevertheless, the evidence demonstrates that the disruption of arginase, an upstream PA pathway regulator, may constitute one of the main causes of AD-related polyamine metabolism; arginase activity is elevated and, as a result, the deficiency of its substrate, arginine, promotes oxidative stress. Moreover, this facilitates the consumption of ornithine to produce putrescine, which further increases PA levels and the PSR to initiate a cycle of neurodegeneration [62].

The most recent research about spermidiIe in AD demonstrates two different perspectives on the involvement of spermidine in mild cognitive impairment (MCI) and AD. On the one hand, one research group demonstrated that in 43 samples from American individuals over 65 years old at the Oregon Alzheimer’s Disease Center, an increase in serum levels of spermidine corresponded to the progression from MCI to AD (Figure 2 and Table 1), suggesting the viability of measuring serum spermidine as a key molecule in AD pathology and as a potential biomarker for AD progression [36]. On the other hand, a study with a rural Chinese population of approximately 3,700 individuals over 35 years old with no history of dementia revealed a non-linear relationship between spermidine and MCI, implying that high levels of spermidine may decrease the burden of MCI (Table 1) [69]. These contradictory reports raise the possibility that factors such as race, diet, and geographic location could change the general perspective on the role of spermidine in AD or MCI.

In recent years, alterations in autophagic flux have been considered a significant factor in the pathology and progression of AD. Spermidine has been found to induce autophagy by inhibiting the negative regulator EP300 (E1A-binding protein p300) [70]. This is particularly important due to EP300’s involvement in aging and age-related diseases, including neurodegeneration in AD. Enhancing autophagy, which aids in the removal of accumulated molecules, may offer protection against the disease or potentially delay the onset of the disease [71,72]. In addition, novel findings in an AD-like mouse model revealed spermidine’s potential to induce autophagy, showing that spermidine promotes the autophagic degradation of the NLRP3 inflammasome, an essential component of the activation of inflammatory signaling pathways; in this scenario, spermidine represents a promising approach to reducing neuroinflammation in an AD mouse model. Moreover, in the same animal model, spermidine demonstrated an increase in the degradation of soluble amyloid beta peptides, but the condition of the plaques and their size were not altered. These results may lead us to debate the effect of spermidine on AD pathology in comparison with its effect on insoluble amyloid beta peptides [73].

A polyamine called spermiIwhich is derived from spermidine, has been identified as a molecule that plays a role in the aggregation of Tau protein. This discovery was made via sophisticated experiments using molecular dynamics simulations. These experiments also showed that spermine has a greater affinity for the phosphorylated form of Tau, which, in turn, alters the structure and distribution of Tau and contributes to the formation of fibrillar deposits in neurons [48]; these findings provide a new outlook for therapeutic development and for understanding the molecular basis of the disease. Further research on PA could lead to the development of interesting therapeutic strategies for neurodegenerative diseases, such as AD. This potential is derived from their natural presence in the human body and the possibility that they could be more effectively tolerated when administered through dietary supplements, either on their own or in combination with other medications.

### 4.2. The Role of PAs in Parkinson’s Disease

The second most common neurodegenerative disorder is Parkinsonߣs disease (PD), which affects more than 1% of the world’s population over 65 years old; estimation studies indicate its prevalence will double by 2030 [74]. This disease leads to alterations in cardinal motor features, slowed movement, rigidity, and tremors, along with other non-motor disturbances such as cognitive decline; these represent the heterogeneity of the symptom burden. As is the case in AD, protein aggregation is a molecular hallmark of PD. Alpha-synuclein (α-synuclein) accumulates in intraneuronal inclusions, causing toxicity and cellular dysfunction [74].

More than a decade ago, it was demonstrated that PD patients with a worse phenotype of the disease showed an increase in putrescine levels in their cerebrospinal fluid (CSF), along with a decrease in spermidine levels (Figure 2 and Table 1) [54]. To date, the molecular role and regulation of the PA levels in a healthy brain or a brain with PD are not well elucidated. Even less is known about how a polyamine’s metabolic changes might impact neurodegenerative diseases. However, a recent study shed light on certain forms of acetylated polyamines, particularly spermidine, which were significantly elevated in the blood serum of PD patients when compared to control groups. Spermidine, a product of the interconversion between spermidine and spermine catalyzed by Spd/Spm acetyltransferase, is now being proposed as a potential biomarker for diagnosing PD and assessing its severity. These findings could also help distinguish PD from other neurological diseases such as AD and progressive supranuclear palsy (PSP) [37].

Regarding protein homeostasis in PD, one interesting molecule is ATP13A2, a lysosomal transporter with five different transmembrane domains. ATP13A2 plays an essential role in maintaining neuronal wellbeing through the regulation of metal ions and the organelle homeostasis of the endoplasmic reticulum, lysosomes, and mitochondria. Interestingly, ATP13A2 also promotes the degradation of polyamines and α-synuclein [75]. The loss of ATP13A2 has been reported in an atypical form of PD, which was determined to cause dysfunction in lysosomal membrane integrity, as it alters the dysfunction of spermidine/spermine exports and consequently promotes the accumulation of α-synuclein [76,77]. Nowadays, efforts to measure the ATP13A2 levels in serum or saliva raise the possibility of it functioning as a potential marker of PD development and complications [78,79]. In general, these findings highlight the importance of studying PA export dysfunction related to ATP13A2 loss in neurodegeneration and through autophagy regulation, which may represent a therapeutic target for delaying these neurodegenerative conditions.

## 5. Polyamines and the Burden of Chronic-Disease Disability

An essential aspect of dealing with the economic burden of age-related diseases is to maintain “successful aging” through the preservation of mobility and physical function so the elderly can live independently and improve their quality of life [80]. Certain health conditions, such as osteoarthritis, sarcopenia, and rheumatoid arthritis, compromise mobility, cause extreme discomfort, and impact quality of life. In this context, new findings related to basic research or adjuvant therapies with natural compounds such as PAs could offer a new perspective on the prevention, diagnosis, and treatment of mobility issues related to aging.

### 5.1. The Role of PAs in Osteoporosis

Among the prevalent age-related diseases, osteoporosis is often at the top of the list. This disease is characterized by a significant decline in bone mass, accompanied by high bone frailty, significantly altering the quality of life of those affected by the disease and increasing mortality, with a high cost for the global economy [81]. Osteoporosis is a complex condition due to its multifactorial etiology, where age, sex, changes in hormone levels, diet, and lifestyle, among other factors, play a pivotal role in the onset of the disease [82]. One of the main challenges with osteoporosis is that it almost never shows symptoms until a fracture occurs, and it is estimated that one in three women and one in five men over 50 years of age have osteoporotic fractures [83,84]. Osteoporosis remains an incurable disease with a large gap in the prevention and management of fractures; this is why new effective strategies are urgently needed to prevent, diagnose, and treat osteoporosis [85].

As noted above, polyamines are essential for life. Regarding bone, mutations in the gene encoding spermine synthase (SMS) result in Snyder–Robinson Syndrome (SRS), an X-linked condition characterized by intellectual disability, seizures, severe osteoporosis, and other symptoms. Loss-of-function mutations in SMS lead to the accumulation of spermidine and decreases in spermine levels, disrupting the strict control of PA homeostasis [86]. A recent nine-year follow-up epidemiological investigation of individuals from 40 to 69 years of age showed that high levels of spermidine and low levels of spermine and putrescine are associated with the risk of osteoporotic fractures, suggesting a central role of SMS in this condition and its possible use as a prognostic marker (Figure 2 and Table 1) [44]. Exogenous polyamines also upregulate the expression of osteogenic genes (e.g., RUNX2, ALP, osteopontin, and OCN) while downregulating the expression of adipogenic genes (e.g., PPAR-γ) [87]. This regulation reduces fat accumulation, could promote the mineralization of the extracellular matrix, and enhances osteogenesis, which is beneficial for bone health [16]. Evidence suggests a role of PA in promoting bone mineralization, but the precise molecular mechanism of its osteogenic potential remains to be elucidated [88]. Other studies have demonstrated that the daily oral supplementation of a diet rich in polyamines, such as polyamine-rich yeast, inhibits osteoclastic activation in mice that underwent ovariectomy (OVX). This suggests that the dietary intake of polyamines can have a positive impact on bone health [89].

### 5.2. The Role of PAs in Osteoarthritis

Osteoarthritis (OA) is a degenerative joint disease associated with aging; it is commonly ignored in terms of its pathophysiology and leads to suffering in the elderly population [90]. To date, there are no effective treatments to prevent, cure, or stop the development of OA. In this context, naturally occurring compounds such as PAs could offer us a better chance of combatting OA. The molecular role of polyamines in joint diseases, such as OA, remains understudied, despite their ubiquitous presence in all tissues, including cartilage. Some results indicate that the PA synthesis pathway could be catalogued as a regulator of chondrocyte differentiation in OA, as it increases the expression of master molecules such as SOX9 [88]. Spermidine has been widely recognized as an inducer of physiological autophagy across different species such as mice, yeast, nematodes, flies, etc. [91]. In recent years, the role of spermidine in human primary chondrocytes has also been described as an inducer of autophagy due to its inhibition of acetyltransferase EP300, an important histone that interacts with key proteins such as LC3 and beclin. Interestingly, spermidine treatments in human and murine chondrocytes showed increases in key chondrogenic markers such as SOX9, aggrecans, and COL2A1, suggesting a potential regenerative response in cartilage [70]. Other studies have reported the chondroprotective and antioxidant effects of spermidine in human samples, improving the autophagic flux in OA [92]. Even though spermidine is known to have anti-inflammatory effects, its effects on OA were not reported until recent years; spermidine has been found to ameliorate OA progression in mouse models by attenuating synovitis, cartilage degeneration, and osteophyte formation through the inhibition of the TNF-α-induced NF-κB/p65 signaling pathway [93]. Another work group demonstrated the ability of PAs to regulate cell differentiation from adipose stem cells to skeletal cells, including chondrocytes; this peculiarity demonstrates that spermine and spermidine can impede oxidative DNA damage indicated by a reduction in γH2AX. These findings offer a promising outlook for therapeutic applications in OA treatment and joint regeneration [94].

All these findings present polyamines, especially spermidine, as natural compounds that could be used for dietary supplementation, serving as adjuvants to ameliorate the burdensome effects of OA.

### 5.3. The Role of PAs in Sarcopenia and Frailty

The roles of polyamines (PAs) and the mammalian target of rapamycin (mTORC1) in regulating protein synthesis and cell growth, especially in skeletal muscle, are well established. Recent research has revealed that PA pathway enzymes demonstrate versatility in responding to various stressors that can promote either muscle growth (hypertrophy) or muscle loss (atrophy). These responses are under the control of mTORC1, highlighting the potential of polyamines in remodeling muscle and aiding in the treatment of muscle diseases, such as sarcopenia [95]. Sarcopenia is recognized as a disease characterized by a significant decline in muscle mass, which entails a substantial decrease in quality of life and increases physical disability; it is accompanied by higher mortality risk, mainly in the elderly [96]. With no currently approved treatment for this disease, the search for new approaches to deal with sarcopenia is a priority.

Novel perspectives on the polyamine spermidine have been gaining attention as potential treatments for sarcopenia, particularly in terms of its association with the androgen receptor (AR). Even though the molecular mechanisms and effects of the androgen receptor (AR) in muscle are not completely understood, novel perspectives regarding its relationship with polyamines, specifically spermidine, are considered interesting insights into the treatment of sarcopenia. Earlier this year, in an AR-KO mouse model that developed the sarcopenia phenotype, a downregulation of gene-related polyamine biosynthesis was demonstrated; there were no significant changes in muscle mass, but an impact on muscle strength was observed in control and middle-aged mice [97]. A metabolomic analysis of skeletal muscle in aged mice demonstrated the significantly decreased expression of the enzymes S-adenosylmethionine decarboxylase and spermine oxidase, which are involved in the synthesis and metabolism of spermine and spermidine, leading to decreases in their levels (Figure 2 and Table 1). Consequently, this could enhance the aging phenotype, such as decreased cell proliferation in primary myoblasts [60]. Other recent studies in mice and rats have shown the upregulation of spermidine/spermine N1-Acetyltransferase 1 when the AR is selectively modulated (by the agonist TEI-SARM2), hypothesizing that the hyperacetylation of polyamines could aid mitochondrial regulation for muscle function [98]. In blood samples from Japanese individuals, the spermine/spermidine ratio was shown to be inversely correlated with the progression of sarcopenia (Figure 2 and Table 1) [40].

All of these findings highlight the importance of studying polyamines as new potential adjuvants for the diagnosis and treatment of sarcopenia.

## 6. Conclusions

As the global healthcare system faces the challenges of age-related diseases and the aging population continues to grow [1], we have an urgent need to find more information about the molecular mechanisms involved in aging. This will allow us to identify possible therapeutic adjuvants and biomarkers that will help prevent, diagnose, and monitor age-related diseases. Because polyamines are essential molecules that play pivotal roles in biochemical and physiological processes in all living organisms, they should be considered molecules that are central to aging [99].

The metabolism of polyamines is tightly regulated, and any disruption of polyamine levels is associated with various diseases, including cancer, where polyamine levels are frequently elevated [30,31]. Furthermore, elevated polyamine levels are also linked to age-related diseases such as PD and AD [67,100]. This contrasts with the conventional perspective, which primarily attributes the decline in polyamine homeostasis to the aging process [101]. This paradox highlights the challenging nature of categorizing the dynamics of polyamines in age-related diseases and their potential use as therapeutic adjuvants. This complexity may arise from treating polyamines as a homogeneous family of molecules with similar roles when, in fact, they can have diverse and even opposing functions in health and disease [102], as well as in different tissues. In general, spermidine (alone or as a dietary supplement) is the best-studied PA and is considered a geroprotective molecule; it has beneficial effects on lifespan and aging in most tissues and species, exhibiting highly versatile mechanisms of action. Nevertheless, its versatility regarding action/functionality can impede our understanding of its therapeutic use, so research on the dose-dependent effects of PAs will improve their possible clinical effectiveness in aging and age-related diseases [103]. To date, there have been very few clinical trials of PAs; those related to aging have evaluated molecular mechanisms such as autophagy and the metabolic response to low-dose spermidine supplementation (ClinicalTrials IDs: NCT05459961 and NCT04823806). Time will tell whether these studies can provide more information about the clinical use of PAs and their effectiveness in aging. All this evidence indicates that, as a next step, we have to gather evidence from individual Pas and PAs as a family to elucidate their effects on therapeutic implementation.

As for age-related diseases that impact disability and mobility, such as osteoarthritis, sarcopenia, and osteoporosis, declines in PA levels have been widely reported. Therefore, strategies to restore the lacking PAs in these age-related diseases via dietary intake could serve as adjuvants in the treatment of these conditions, consequently improving the futures and quality of life of the elderly.

Nowadays, there is an obvious need for biomarkers that lead to the detection of many diseases in the elderly; in this context, metabolomics has been a valuable tool for measuring the perturbation of many metabolites in different biofluids such as serum [29], including PAs. Metabolomics has positioned PAs as central mediators of aging and as new biomarkers for diagnosing and monitoring age-related diseases. Even if metabolomics has the potential to decipher metabolite profiles in aging and age-related diseases, the challenge of providing conclusive information about useful biomarkers is yet to be resolved.

## Figures and Tables

**Table 1:** Polyamine impairment and age-related diseases in humans.

Disease	Type of Sample	Polyamine Impairment Effect	Ref.
**Alzheimer’s Disease**	Temporal cortex and occipital cortex tissue	- Spermidine was increased (70%) and putrescine was decreased (28%) in temporal cortex. - Spermine was reduced 35% in occipital cortex.	[45]
	Cortical surface tissue	- Higher levels of S-adenosyl methionine and spermidine. - Polyamine-associated gene transcripts were significantly dysregulated.	[46]
	Brain tissue	- Ornithine decarboxylase activity was significantly increased in temporal cortex (76%) and reduced in occipital cortex (70%).	[47]
		- Spermidine and acetyl-spermidine were increased. - Putrescine, spermine, and acetylated spermine levels were also elevated.	[48]
		- Spermidine and spermine levels were significantly increased. - Putrescine, Ac-SPD, and Ac-SPM were increased. - Ornithine levels did not change significantly.	[49]
		- Polyamine-associated gene transcripts, including ODC (FC = 2.15), ASS1 (FC = 1.78), and AZIN2 (FC = 2.43), were significantly increased.	[50]
	Plasma and CSF	- Polyamine metabolism was one of the most affected pathways in plasma of AD patients compared with mild cognitive impairment patients.	[51]
	Serum	- Mild cognitive impairment patients showed significantly higher serum spermidine levels.	[36]
**Parkinson’s Disease**	Brain tissue	- Increased polyamine levels.	[52]
		- Putrescine levels were decreased. - Spermidine levels were decreased in putamen.	[53]
	CSF	- Polyamines were altered: N1-acetylcadaverine (1.40 ±0.70), putrescine (0.2 ± 0.02), cadaverine (3.34 ± 1.03), N8-acetylspermidine (0.38 ± 0.14), spermidine (0.07 ± 0.01).	[54]
	CSF and serum	- N-acetylcadaverine and N-acetylputrescine showed a significant change.	[55]
	CSF and red blood cells	- Increased levels of spermidine and spermine. - Decreased levels of putrescine in red blood cells. - Higher concentrations of putrescine, cadaverine, N1-acetyl-Cad, and N1-acetyl-Spd. - Lower levels of Spd in CSF.	[56]
	Serum	- N8-acetyl spermidine was increased in patients with rapid-progressor phenotype compared to both control subjects and slow progressors.	[57]
		- Ornithine metabolite was increased.	[58]
	Plasma	- Elevated N8-AcSpd (ratio: 1.44) and N-acetylputrescine (ratio: 1.20). - Spm/Spd ratio in blood was decreased.	[37]
**Osteoporosis**		- Low spermine and putrescine levels were associated with individuals with osteoporotic fractures.	[44]
**Osteoarthritis**	Serum	- Spermine/spermidine ratio was decreased (0.898 + 0.227 vs. 1.060 + 0.279).	[39]
**Sarcopenia**	Whole blood	- Spermidine levels were higher. - Spermine/spermidine ratio was lower.	[40]
	* Mouse skeletal muscle	- A downregulation of two of the key encoding enzymes involved in polyamine biosynthesis (Odc1 and Amd1) was observed (2.65- and 1.97-fold, respectively) in 24-month-old muscle.	[59]
		- Spermidine and spermine were significantly decreased. - The mRNA level of ornithine decarboxylase increased (1.4-fold). - RNA of S-adenosylmethionine decarboxylase and spermine oxidase decreased (0.3-fold and 0.2-fold, respectively).	[60]

* Refers to studies using murine model.

## Data Availability

Not applicable.

## Abbreviations

α-syn	Alpha-synuclein
AD	Alzheimer’s disease
ADC	Arginine decarboxylase
ATP13A2	ATPase cation transporting 13A2
BMI	Bone mass index
CNS	Central nervous system
CSF	Cerebrospinal fluid
COL2A1	Collagen type II alpha 1 chain
DHS	Deoxyhypusine synthase
DOHH	Deoxyhypusine hydrolase
EP300	E1A-binding protein P300
elF5A	Eukaryotic translation initiation factor 5A
GPC1	Glypican 1
	
MCI	Mild cognitive impairment
MTA	5ߣ-methylthioadenosin
NMDA	N-Methyl-D-aspartate
NLRP3	NLR family pyrin domain containing 3
OCT-1-3	Organic cation transporters 1, 2, and 3
ODC	Ornithine decarboxylase
OA	Osteoarthritis
OVX	Ovariectomy
PD	Parkinson’s disease
PAO	Polyamine oxidase
PA	Polyamines
PUT	Putrescine
PSP	Progressive supranuclear palsy
PSR	Polyamine stress response
dcAdoMet	S-adenosylmethionine
SRS	Snyder–Robinson Syndrome
SLC3A2	Solute Carrier Family 3 (Amino Acid Transporter Heavy Chain)
SPD	Spermidine
SMS	Spermidine synthase
SPM	Spermine
SSAT	Spermine/spermidine N1-acetyltransferase
SOX9	SRY-box transcription 9
WHO	World Health Organization
